# Assessment of Exercise Testing after Repair of Tetralogy of Fallot

**DOI:** 10.5402/2012/324306

**Published:** 2012-09-02

**Authors:** A. A. Kotby, H. M. Elnabawy, W. M. El-Guindy, R. F. Abd Elaziz

**Affiliations:** Pediatric Department, Faculty of Medicine, Ain Shams University, Cairo 11231, Egypt

## Abstract

Tetralogy of Fallot (TOF) is the most common form of cyanotic congenital heart disease. The aim of this study was to examine the exercise performance of young patients following the repair of TOF and to assess the influence of different variables related to the surgical repair on exercise testing. This study was conducted on 21 patients (16 males and 5 females) operated on for TOF compared to 15 healthy age- and sex-matched control children. The patients' median age at time of the study was 8 years (range 5–13 years) while age at surgical repair was 5 ± 2.1 years (range 2–10 years). Patients were subjected to 2D and color Doppler echocardiographic examination. Treadmill exercise stress testing was performed for all subjects according to modified Bruce protocol. The resting ECGs of all patients revealed normal sinus rhythm and RBBB. Cases had lower exercise capacity when compared to control subjects and those with aortopulmonary shunt showed significantly lower exercise performance when compared to those without aortopulmonary shunt. In conclusions, exercise tolerance after total correction of TOF is slightly impaired on short-term followup with more affection among patients with previous aortopulmonary shunts. The present study did not reveal any serious ventricular arrhythmia.

## 1. Introduction

Tetralogy of Fallot (TOF) is the most common form of cyanotic congenital heart disease. Impairment in exercise tolerance after total repair of tetralogy of Fallot has been frequently reported and speculated to be due to variable causes including residual right ventricular outflow tract (RVOT) obstruction, branch pulmonary artery stenosis, pulmonary insufficiency, pulmonary pathology, and chronotropic incompetence [1]. Pulmonary regurgitation (PR) has been shown to be related to the use of transannular patch during RVOT reconstruction and aggressive infundibulectomy involving the pulmonary valve annulus. Adverse effects of PR include progressive dilatation of RV, reduced exercise capacity, arrhythmia, and sudden death [2]. A number of children have premature ventricular beats after repair of the tetralogy of Fallot. These beats are of concern in patients with residual hemodynamic abnormalities; 24-hr electrocardiographic (Holter) monitoring studies should be performed to be certain that occult short episodes of ventricular tachycardia are not occurring. Exercise studies may be useful in provoking cardiac arrhythmias that are not apparent at rest [3]. The aim of this study was to assess the exercise performance of young patients following the repair of Tetralogy of Fallot and to assess the influence of different variables related to the surgical repair on exercise testing.

## 2. Subjects and Methods

This study was carried out at the Children's hospital, Pediatric cardiology department, Ain Shams University in the period from March 2008 to February 2010. It included 21 patients operated on for tetralogy of Fallot compared to 15 healthy age- and sex-matched children. All patients underwent total correction for TOF at ages ranging between 2 and 10 years. Total correction was performed by closing the VSD and reconstruction of the RVOT via either transatrial-transpulmonary or right ventriculotomy approaches. Seven patients (33.3%) had palliative aortopulmonary shunt prior to total correction. Fourteen patients (66.7%) needed transannular patch for RVOT reconstruction during total correction. All patients were subjected to twelve-lead ECG to comment on rate, rhythm, *P* wave, axis, QRS duration, bundle branch block, and chamber enlargement. M mode, two-dimensional, and color Doppler echocardiographic examination using GE Health Care Ultrasound Vivid 7 was done for all patients to comment on pulmonary valve regurgitation or residual stenosis, branch pulmonary arteries, right ventricular size, and function, residual VSD and leak across, movement of IVS, left ventricular function, and other associated cardiac anomalies. Exercise stress testing was performed by using a Schiller MTM-1500 Treadmill using the modified Bruce protocol.

## 3. Results

The resting ECGs of all patients revealed normal sinus rhythm and RBBB, 16 patients with complete RBBB, and 5 patients with incomplete RBBB. QRS durations were prolonged but less than 180 msec in 19 patients, and more than 180 msec in only 1 patient. There was no conduction disturbance noticed in the control group. Echocardiographic examination showed variable degrees of pulmonary regurge in 20 patients (95%); severe PR in 12 patients, moderate PR in 2 patients, and mild PR in 6 patients. There was abnormal right ventricular dilation in 5 patients (23.8%) detected by 2D Echo. Residual VSD was detected in 2 patients and branch pulmonary artery stenosis in 5 patients. Control subjects have completely normal echocardiography. Three patients (14.2%) developed exercise-induced uniform infrequent PVCs during stress testing. Sustained ventricular tachycardia was not detected in any patient. While infrequent PVCs developed in 2 control subjects (13.3%). Only one patient (4.7%) of the study group developed exercise-induced complete heart block (CHB) as detected by stress testing. There was no statistical significant difference between cases and control as regards the work time. Cases showed lower work stage and time, but the difference did not reach a significant level *P* = 0.10, 0.22. While they showed significantly lower max SBP, max HR, max METs, and % of METs when compared to control *P* < 0.05 as shown in Table 1.

Cases with aortopulmonary shunt showed significantly lower work time, work stage, and Max METs when compared with those without aortopulmonary shunt *P* < 0.05. as shown in Table 2.

## 4. Discussion

Surgical correction of TOF is directed at relieving all possible sources of right ventricular outflow tract obstruction. If anatomically and surgically possible, pulmonary valve function is preserved by avoiding a transannular patch. The ventricular septal defect may be closed from either a ventricular or atrial approach [4]. Palliative aortopulmonary shunt procedures are performed prior to total correction to increase pulmonary blood flow in severely cyanotic infants [5]. Numerous 20 to 30 years follow-up studies have documented the excellent clinical results of surgical repair of TOF. However, the nature of the repair leaves each patient with some degree of excessive hemodynamic burden because of residual defects, valvular abnormalities, or myocardial factors [4]. Resting ECGs of all patients revealed normal sinus rhythm and RBBB, 16 patients with complete RBBB, and 5 patients with incomplete RBBB, whereas no conduction disturbances were shown in the control group. This was in accordance to Sarubbi et al. [6] who assessed the exercise capacity in young patients after total repair of Fallot tetralogy and found that all the patients presented complete RBBB, whereas no conductance disturbances were shown in the control group. Patients with repaired TOF frequently have right bundle-branch block with greatly prolonged QRS duration, usually attributed to the effects of cardiac surgery and this electric abnormality has been recognized not only as a risk factor for sudden cardiac death in these patients but also as a contributor to RV dysfunction [7]. In our study pulmonary regurgitation was observed in 20 patients (95%), 12 of them (57%) had severe PR and 8 (38%) had mild-to-moderate PR. This is in accordance to Eyskens et al. [8] who found that all patients had variable degrees of pulmonary regurgitation on standard echocardiography after total correction of TOF. Pulmonary regurgitation has been attributed to the use of transannular patch during RVOT reconstruction and aggressive infundibulectomy involving the pulmonary valve annulus. Adverse effects of PR include progressive dilatation of RV, reduced exercise capacity, arrhythmia and sudden death [2]. Our study showed no statistical significant difference between cases and control as regards the work time. Cases showed lower work stage and time but the difference did not reach a significant level. As reported by Van der Cammen-van Zijp et al. [9] the maximal work time served as a criterion of exercise capacity when maximum O_2_ consumption cannot be measured. This result showed that exercise capacity in patients undergoing surgical repair was generally good as compared with matched control subjects. This was in accordance to Pietrucha and Rudzinski [10] who assessed the exercise capacity in 23 patients after surgical correction of TOF (21 males and two female) with mean age 14.9 ± 2.9 years and 27 patients without any cardiac disorder (19 boys, 8 girls) age- and sex-matched and found that workload achieved by patients after tetralogy of Fallot correction was comparable to healthy subjects. Moreover Mahle et al. [11] found that intermediate-term exercise performance in patients who underwent primary complete repair of TOF in early childhood was nearly normal. Several previous studies observed an association between pulmonary regurgitation and exercise impairment in the form of decreased exercise time, VO_2_ max and maximum achieved METs when compared with normal control subjects during exercise stress testing. In our study, this relationship was not detected. However, our results are similar to Samman et al. [12] who were unable to demonstrate a relationship between the degree of pulmonary regurgitation and exercise capacity in patients with repaired tetralogy of Fallot and ascribed these contradictory finding to the different methods used to measure the degree of pulmonary regurgitation in these studies in comparison with their study. Also our results are in accordance to Geva et al. [13] who found that PR degree was not correlated with RV function or exercise capacity after TOF repair. Rathore et al. [14] found no correlation between the presence or degree of pulmonary regurgitation and its effect on exercise capacity after TOF repair. As shown in our study, there was no relation between the degree of pulmonary regurge and the exercise capacity in short term followup. As impaired exercise tolerance after TOF repair as detected by exercise stress testing (decreased work time, work stage, max HR, and max achieved METs when compared with control subjects) is multifactorial in origin; patients with severe pulmonary regurge may have cardiovascular compensation with good chronotropic response to exercise while those with mild or no PR may have other causes that impair their exercise tolerance as chronotropic incompetence and lack of physical fitness. In the present study, cases with previous aortopulmonary shunt showed significantly lower exercise indices when compared to those without previous aortopulmonary shunt. This was unlike Sarubbi et al. study [6] who found that prior aortopulmonary shunt procedures were not associated with reduced exercise performance after TOF repair. Impaired exercise performance in the patients with previous aortopulmonary shunts might be due to sever TOF that necessitated the performance of a shunt. Moreover, palliation by means of an aortopulmonary shunts that had been initially performed, leading to changes in the pulmonary vascular system, which is pathologically altered and can in turn, negatively affect exercise capacity [15]. We discovered that only one patient (4.7%) of our study group developed exercise-induced complete heart block (CHB) as detected by stress testing. Surprisingly, the patient had poor unexplained weight gain. The CHB revealed by his exercise test probably contributed to the growth impairment of that child. Andersen et al. [16] reported that 1% of their patients developed CHB 26 years after TOF repair.

## 5. Conclusion 

Exercise tolerance after total correction of Fallot tetralogy is slightly impaired on short-term followup. Thus abnormal cardiac function and haemodynamic abnormalities secondary to PR and residual defects appear after longer periods of followup. Patients with previous aortopulmonary shunts showed lower exercise performance when compared to those without previous aortopulmonary shunt. The present study did not reveal any serious ventricular arrhythmias since it is seen in those patients with the longest period of follow up. Postoperative exercise testing in patients with TOF can unmask a complete heart block that was not elicited by the standard 12-lead ECG recording.

## Figures and Tables

**Table 1:** Comparison between patients and control as stress testing regarding exercise.

	Control		Cases		*Z*	*P*	Sig.
	Median	IQR	Median	IQR			
Work time (min)	12.40	9.57–15.23	11.00	9.34–13.28	1.22	0.22	NS
Work stage	5.00	4.00–6.00	4.00	4.00-5.00	1.66	0.10	NS
Max HR (bpm)	176.00	158.00–186.00	155.0	136.00–168.50	2.30	0.02	S
% of target HR	89	82–96	79	71–91	2.10	0.03	S
Max SBP	145.00	138.00–160.00	130.00	120.00–145.00	2.13	0.03	S
Max DBP	80.00	70.00–85.00	80.00	75.00–83.50	0.20	0.84	NS
Max METs	10.10	7.00–11.60	7.00	5.80–10.00	2.63	0.009	S
% of METs	55.00	45.00–71.00	31.00	27.00–47.50	3.05	0.002	S

HR: heart rate, SBP: systolic blood pressure, DBP: diastolic blood pressure, MET: metabolic equivalent.

**Table 2:** Comparison between patients with and without aortopulmonary shunt with regard to exercise stress testing.

	Aortopulmonary shunt						
	No (14)		Yes (7)		*z*	*P*	Sig.
	Median	IQR	Median	IQR			
Work time (min)	12.30	10.57–14.06	9.35	2.27–10.15	2.76	0.006	S
Work stage	5.00	4.00-5.00	4.00	1.00–4.00	2.76	0.006	S
Max HR (bpm)	156.50	136.00–171.25	146.00	136.00–169.00	0.37	0.71	NS
% of target HR	83	72–94	76	70–87	0.71	0.48	NS
Max SBP	142.50	120.00–151.25	125.00	120.00–135.00	1.58	0.11	NS
Max DBP	80.00	73.75–85.00	80.00	75.00–80.00	0.85	0.40	NS
Max METs	7.00	7.00–10.10	4.60	2.30–7.00	2.74	0.006	S
% of METs	44.50	29.75–50	24	16–30	2.99	0.003	S

HR: heart rate, SBP: systolic blood pressure, DBP: diastolic blood pressure, MET: metabolic equivalent.

## References

[1] Anji T, Lee KJ, Hamilton R, Morrow WR, McCrindle BW (2001). Exercise capacity after repair of tetralogy of Fallot in infancy. *American Journal of Cardiology*.

[2] Bouzas B, Kilner PJ, Gatzoulis MA (2005). Pulmonary regurgitation: not a benign lesion. *European Heart Journal*.

[3] Kliegman RE, Behrman RE, Jenson HB, Bernstein (2008). Cyanotic congenital heart lesions. *Nelson Textbook of Pediatrics*.

[4] Siwik E, Erenberg F, Zahka K, Goldmuntz E, Allen HD, Clark EB, Gutgessell HP (2008). Tetralogy of Fallot. *Moss and Adam's Heart Disease in Infants, Children and Adolescents*.

[5] Park A, Myung K (2008). Cyanotic congenital heart disease, tetralogy of Fallot. *Pediatric Cardiology for Practitioners*.

[6] Sarubbi B, Pacileo G, Pisacane C (2000). Exercise capacity in young patients after total repair of tetralogy of Fallot. *Pediatric Cardiology*.

[7] Derek G, Uebing A, Gibson V, Gerhard P, Michael A (2007). Right ventricular mechanics and QRS duration in patients with repaired tetralogy of Fallot: implications of infundibular disease. *Circulation*.

[8] Eyskens B, Stephen C, Claus P, Gewillig M, Bogaert J, Mertens L (2010). The influence of pulmonary regurgitation on regional right ventricular function in children after surgical repair of tetralogy of Fallot. *European Journal of Echocardiography*.

[9] van der Cammen-van Zijp MHM, Ijsselstijn H, Takken T (2010). Exercise testing of pre-school children using the Bruce treadmill protocol: new reference values. *European Journal of Applied Physiology*.

[10] Pietrucha BJ, Rudzinski A (2008). Exercise capacity in children after surgical correction of tetralogy of Fallot. *European Journal of Heart Failure Supplements*.

[11] Mahle WT, McBride MG, Paridon SM (2002). Exercise performance in tetralogy of Fallot: the impact of primary complete repair in infancy. *Pediatric Cardiology*.

[12] Samman A, Schwerzmann M, Balint OH (2008). Exercise capacity and biventricular function in adult patients with repaired tetralogy of Fallot. *American Heart Journal*.

[13] Geva T, Sandweiss BM, Gauvreau K, Lock JE, Andrew M (2004). Factors associated with impaired clinical status in long-term survivors of tetralogy of Fallot repair evaluated by magnetic resonance imaging. *Journal of the American College of Cardiology*.

[14] Rathore KS, Agrawal SK, Kapoor A (2006). Restrictive physiology in tetralogy of Fallot: exercise and arrhythmogenesis. *Asian Cardiovascular and Thoracic Annals*.

[15] Norozi K, Gravenhorst V, Hobbiebrunken E, Wessel A (2005). Normality of cardiopulmonary capacity in children operated on to correct congenital heart defects. *Archives of Pediatrics and Adolescent Medicine*.

[16] Andersen H, Marc R, Tsang VT, Elliott MJ, Robert H (2006). Is complete heart block after surgical closure of ventricular septum defects still an issue?. *Annals of Thoracic Surgery*.

